# Population Approaches Targeting Metabolic Syndrome Focusing on Japanese Trials

**DOI:** 10.3390/nu11061430

**Published:** 2019-06-25

**Authors:** Hitoshi Nishizawa, Iichiro Shimomura

**Affiliations:** Department of Metabolic Medicine, Graduate School of Medicine, Osaka University, 2-2-B5, Yamada-oka, Suita, Osaka 565-0871, Japan; ichi@endmet.med.osaka-u.ac.jp

**Keywords:** atherosclerotic cardiovascular disease, visceral fat accumulation, universal public health screening program, health check-up, health guidance, city planning

## Abstract

The clinical importance of assessment of metabolic syndrome lies in the selection of individuals with multiple risk factors based on visceral fat accumulation, and helping them to reduce visceral fat. Behavioral modification by population approach is important, which adds support to the personal approach. The complexity of visceral fat accumulation requires multicomponent and multilevel intervention. Preparation of food and physical environments could be useful strategies for city planners. Furthermore, actions on various frameworks, including organizational, community, and policy levels, have been recently reported. There are universal public health screening programs and post-screening health educational systems in Japan, and diseases management programs in Germany. Understanding one’s own health status is important for motivation for lifestyle modification. The U.S. Preventive Services Task Force recommends that primary care practitioners screen all adults for obesity and offer behavioral interventions and intensive counseling. Established evidence-based guidelines for behavioral counseling are needed within the primary care setting.

## 1. Introduction

**Figure 1:**
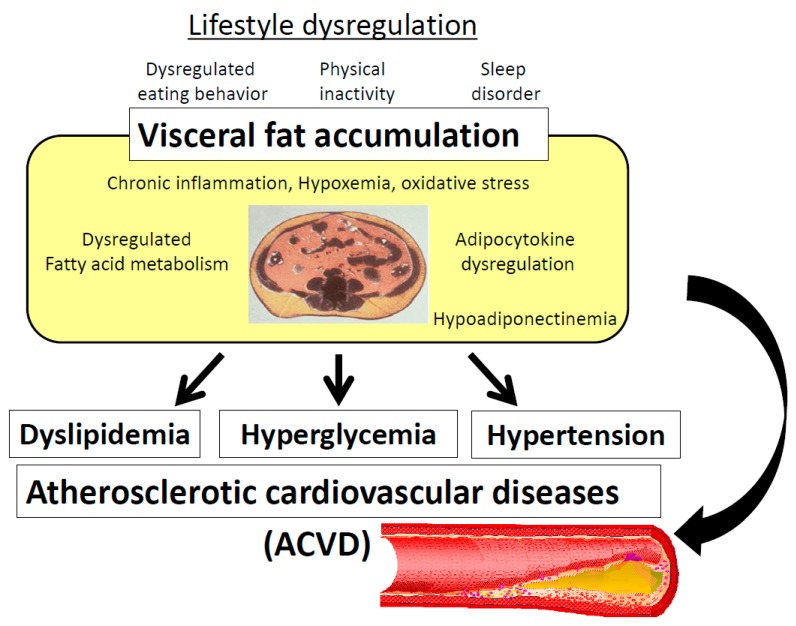
Concept of metabolic syndrome.

**Figure 2:**
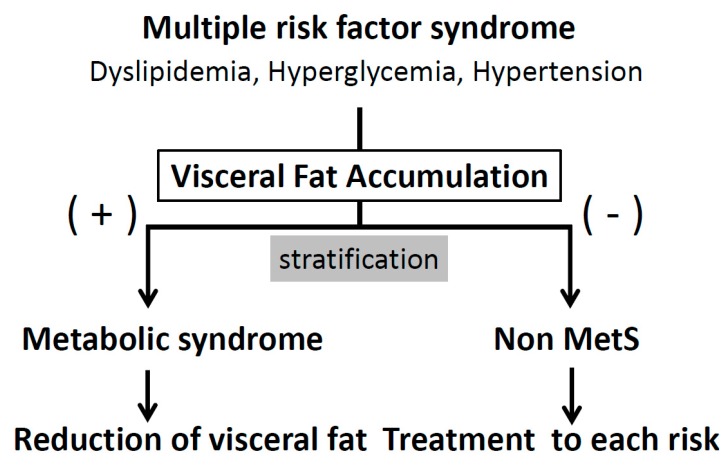
Management of multiple risk factor syndrome for prevention of atherosclerotic cardiovascular diseases (ACVD); “Metabolic syndrome (Mets)-oriented approach”.

Visceral fat accumulation is associated with glucose intolerance, dyslipidemia, hypertension, and atherosclerotic cardiovascular diseases (ACVD), conceptualized as metabolic syndrome (Figure 1). Several definitions of metabolic syndrome have been used worldwide. Abdominal obesity is one of the risk factors in the harmonized criteria by AHA (American Heart Association), IDF (International Diabetes Federation), NHLBI (National Heart, Lung, and Blood Institute), and other organizations (2009) [1], while in the original criteria of IDF (2005) [2] and the Japanese criteria [3,4], abdominal obesity or visceral fat accumulation is an essential component of metabolic syndrome. The latter criteria consider visceral fat accumulation as the basal pathogenic component of metabolic syndrome. An important aspect of the diagnosis of visceral fat-based metabolic syndrome is to select subjects with multiple risk factors based on visceral fat accumulation, and enroll them in health educational programs conducted at health check-up and medical facilities. The cornerstone of effective improvement of multiple risk factors for ACVD is the reduction of visceral fat (Figure 2).

The cause of worldwide obesity and visceral fat accumulation is multifactorial at multiple levels, including food environment, physical activity levels, and policies. One of the important approaches to reduce visceral fat involves behavioral modification of individuals (personal approach), such as modifications to dietary and physical habits. Another is the population approach, which provides support to the personal approach, and could be even more important.

## 2. Obesity and Obesity Disease

Obesity is a state of excess body fat accumulation in individuals, and body mass index (BMI) is used as an index of obesity. Obesity is defined as BMI ≥ 30 kg/m^2^ in Europe and the United States, and ≥ 25 kg/m^2^ in Japan, where there are fewer obese individuals [5,6,7]. Obesity, per se, is not always clinically necessary to be subjected to aggressive medical intervention. On the other hand, “obesity disease” is defined in Japan as visceral fat obesity (BMI ≥ 25 kg/m^2^ plus visceral fat area ≥ 100 cm^2^), or obesity (BMI ≥ 25 kg/m^2^) with obesity-related complications, such as metabolic or orthopedic disorders that require weight reduction for their improvement [7]. Therefore, “Obesity disease” should be dealt with as a clinical condition requiring medical intervention.

## 3. Visceral Fat and Subcutaneous Fat

It is well known that obesity is accompanied by glucose intolerance, dyslipidemia, and hypertension. Following the spread of the Western diet and motor vehicles, the prevalence of obesity and type 2 diabetes has increased worldwide [5,6]. East Asians, including Japanese, are more easily affected by metabolic disorders even with a mild degree of obesity, compared with Europeans and Americans. On the other hand, such disorders are not always accompanied by massive obesity. These disorders cannot be explained by the absolute value of BMI, and therefore understanding of body fat distribution is considerably important [8]. Analysis of body fat distribution using abdominal computed tomography (CT) has demonstrated that visceral fat obesity poses a higher risk for metabolic disorders and ACVD than subcutaneous obesity [9]. A recent Japanese study involving subjects who underwent health checks concluded that visceral fat area (VFA), but not subcutaneous fat area (SFA), correlated positively with the number of cardiovascular risk factors [10]. The same study also demonstrated that the mean number of risk factors exceeded one at 100 cm^2^ of VFA, both in males and females. Therefore, the cutoff value of visceral fat accumulation was set to 100 cm^2^ in Japan [10].

## 4. BMI and Visceral Fat Area

**Figure 3:**
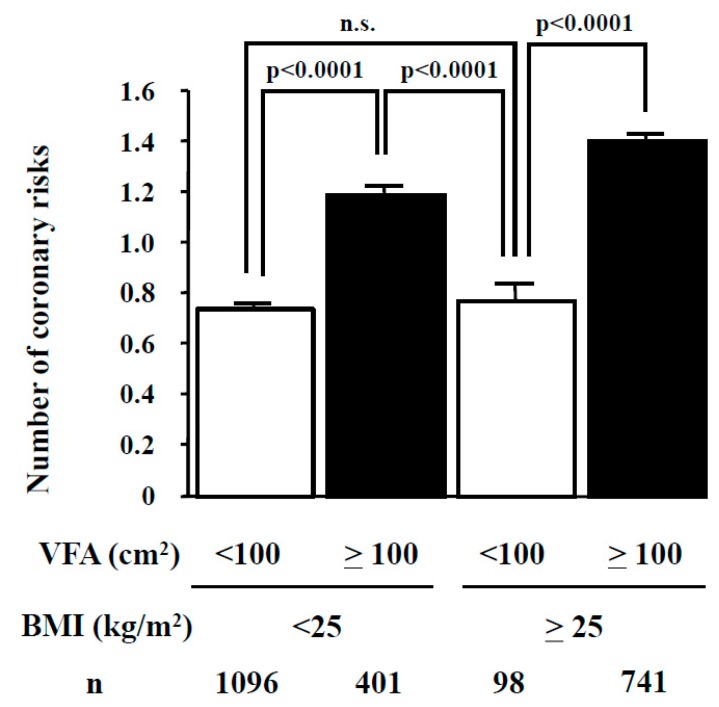
Relationship between number of metabolic risk factors and body fat distribution. Subjects were divided according to their BMI (cutoff value 25 kg/m^2^) and VFA (cutoff value 100 cm^2^). Data are mean ± SEM. BMI = body mass index; VFA = visceral fat area;. Data from Diabetes Care 2007; 30: 2392-94 [11], by permission of American Diabetes Association.

The BMI and VFA vary considerably among individuals, especially in males. In a study of Japanese male employees (mean age 48.0 ± 10.5 years, ±SD), 26.8% (*n* = 401/1497) of normal weight subjects (BMI < 25 kg/m^2^) had visceral fat accumulation (VFA ≥10 0cm^2^) (Figure 3) [11]. Irrespective of BMI, the mean number of metabolic risk factors in the subjects with visceral fat accumulation was significantly higher than those without (solid bars in Figure 3). Also, a study from the United Kingdom reported a relatively high mortality rate for individuals with central obesity, despite having normal weight (BMI <25 kg/m^2^) [12]. Interestingly, reduction of body weight and VFA is closely associated with improvement of metabolic risk factors, such as diabetes, dyslipidemia, and hypertension [11,13]. These data suggest that assessment of visceral fat accumulation is important for selection of individuals who should avoid over-nutrition.

## 5. Clinical Significance of Metabolic Syndrome in Atherosclerotic Cardiovascular Diseases

It is important to take measures against individual risk factors, such as hypertension, smoking, and hypercholesterolemia, to prevent atherosclerotic cardiovascular diseases (ACVD). Since the 1990s, multiple risk factor syndrome and metabolic syndrome have been the focus of attention as residual risks, in which dysregulation of glucose and lipid metabolism, hypertension, and obesity coexisted in each individual [1,14,15]. Among them, the concept of metabolic syndrome was selected in the original criteria of IDF (2005) along with in Japan, stating that visceral fat accumulation is the basis of the pathogenesis of atherosclerosis complicated with dysregulation of glucose and lipid metabolism and elevated blood pressure (Figure 1). There are two types of arteriosclerosis—atherosclerosis that affects relatively large vessels, such as coronary arteries and middle cerebral arteries, and arteriosclerosis that affects relatively small vessels, such as cerebral perforating arteries. Atherosclerosis is the pathological process underlying myocardial infarction and cerebral thrombosis, whereas arteriosclerosis is that of cerebral hemorrhage and lacunar infarction. In Europe and the United States, atherosclerosis based on dyslipidemia and metabolic syndrome is more frequent due to over-intake of dietary fat. On the other hand, in East Asia, arteriosclerosis based on hypertension has been the predominant type due to over-intake of dietary salt [16]. Recently, even in East Asia and Japan, visceral fat based-metabolic syndrome has been increasing. However, hypertension and hypercholesterolemia should be important therapeutic targets in any clinical management program designed to prevent ACVD independent of the metabolic syndrome.

## 6. Pathogenesis of Visceral Fat Accumulation

Visceral fat is the adipose tissue that adheres and accumulates in the mesenterium and omentum, and acts as transient energy reservoir from gut to liver through the portal vein. In fasting and starvation, lipolysis efficiently occurs in visceral fat resulting in supply of free fatty acids and glycerol to hepatocytes. However, through excessive lipolysis in the accumulated visceral fat, large amounts of free fatty acids and glycerol overflow into the liver, resulting in the dysregulation of lipid metabolism and gluconeogenesis [17]. Although the weight of adipose tissue accounts for about 15–20% of total body weight, that of obese subjects reaches up to 30–50%. Therefore, the massive adipose tissue considerably affects the pathogenic condition of individuals.

Adipocyte precursor cells in visceral adipose tissue are relatively difficult to differentiate or proliferate compared to those in the subcutaneous adipose tissue [18]. Thus, visceral adipose cells, in parallel to over-nutrition, are considered hypertrophic adipocytes. Since the number of adipocytes can increase only during childhood and adolescence [19], over-nutrition in adulthood induces hypertrophy of visceral adipose cells, which are affected by hypoxia and inflammation, complicating the production of oxidative stress and dysregulation of adipocytokines and adipokines, such as hypoadiponectinemia. This is the pathogenic basis of visceral fat accumulation associated with metabolic syndrome (Figure 1) [20,21].

Fat distribution varies considerably between males and females and also among different ethnic groups. In Asian individuals, VFA is relatively larger than SFA [22,23]. This is probably related to genetic (ethnic) differences in the proliferative potential of subcutaneous adipose precursor cells, as well as differences in the duration of over-nutrition. Therefore, the susceptibility of Asian individuals to the metabolic syndrome could be higher, with visceral fat accumulation even in lower BMI relative to Europeans and Americans.

## 7. Population Approaches Targeting the Metabolic Syndrome

**Figure 4:**
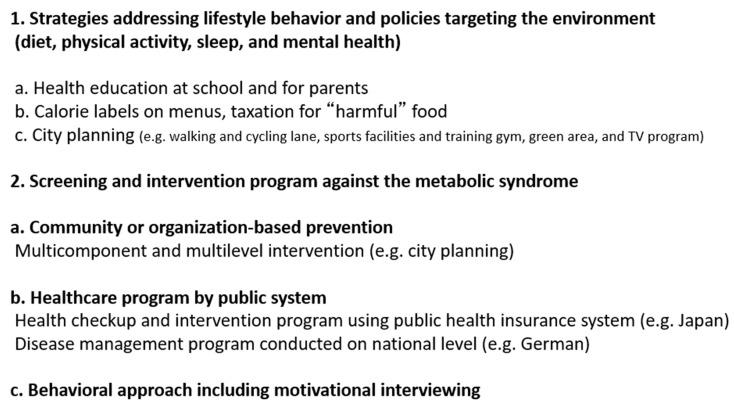
Population Approaches targeting the metabolic syndrome.

There is a need to reduce accumulated visceral fat in subjects with metabolic syndrome, rather than treat each metabolic risk factor with medications. To achieve this aim, it is important to provide health education about healthy diet and physical exercise to these individuals (personal approach). Next, improvement of various metabolic risk factors can be achieved through reduction of accumulated visceral fat [11,13]. Therefore, for assessment of the metabolic syndrome, it is clinically important to identify individuals with large amounts of visceral fat during medical or health check-ups, who are affected, or are supposed to be, by multiple metabolic disorders, even if each disorder is mild. Moreover, it is important to enroll the subjects with metabolic syndrome into the health education system. We will focus here on population approaches to combat metabolic syndrome (Figure 4), as individual programs on lifestyles, such as diet and physical exercise, are discussed in other chapters of this review and have also been described previously [24].

## 8. Strategies Addressing Lifestyle Behavior and Policies Targeting the Environment (Diet, Physical Activity, Sleep, and Mental Health)

For lifestyle modification, it is first important to have a clinical understanding of one’s own health status. For this aim, health check-ups followed by health guidance is a good approach. Recent studies have provided evidence for the role of pictorial presentation of silent atherosclerosis in the prevention of cardiovascular diseases and its usefulness in reducing the low adherence to medications and lifestyle modification [25]. Therefore, scientific understanding of one’s own health is the most important factor for motivation towards lifestyle modification. Once subjects are motivated to improve their lifestyle to reduce accumulated visceral fat, health promotion strategies, which address lifestyle behavior and policies targeting the environment, are potentially effective in the prevention of visceral fat accumulation.

Since dieting and disordered eating behaviors during childhood and adolescence are considered to continue to be present among young adults [26], some strategies were found to be useful, such as health education on nutrition for students and their parents in school and provision of healthy food at school (Figure 4) [27,28]. At the population level, public policies and economic strategies are important to improve food and physical environments. For example, calorie and nutritional information on food menus and packaging are useful for individuals who care about dietary modification and also to avoid harmful food components, such as saturated fatty acids and excess salt. In some countries, higher taxes have been enforced on harmful foods, such as fast foods, unhealthy fats, and sugar-sweetened drinks (Figure 4) [29].

High physical activity is reported to be associated with low risk of mortality and cardiovascular disease [30], and diet-plus-exercise was more effective in weight loss than diet-only interventions [31]. Strategies that encourage individuals to exercise are important. Walking and cycling can serve for both transportation and recreational purposes, and both can reduce motor vehicle dependency. Therefore, it should be important to support lifestyle choices through city planning, such as preparation for walking, running, and cycling lanes (Figure 4) [32]. In general, aerobic exercise several days per week (total: 150 min/week) has been recommended in physical activity guidelines [33]. However, it was recently reported that there was no significant difference in the likelihood of metabolic syndrome in the general population between frequently active participants (≥5 days per week) and infrequently active participants (1–4 days per week) after adjustment for total weekly moderate-to-vigorous physical activity [34], suggesting that only weekend exercise could be effective in preventing metabolic syndrome if sufficient weekly physical activity was performed. Preparation of sports gymnasiums, playing fields, and parks that are easily accessed by citizens are useful strategies that should be instituted in city planning.

Sleep is considerably associated with eating behaviors and physical activity. Insufficient sleep has been reported to be associated with dysregulation of leptin and ghrelin [35], resulting in hyperphagia and physical inactivity due to sleepiness. Moreover, a late bedtime with shorter sleep time were reported to be closely associated with weight gain and visceral fat accumulation [36,37]. To facilitate more sleeping hours, many communities have attempted to change TV programming [32]. Since control of psychological status is important to continue lifestyle modification, green areas in communities are useful for stress management (Figure 4) [28,32].

## 9. Screening and Intervention Program Against the Metabolic Syndrome

### 9.1. Community or Organization-Based Prevention

The complexity of visceral fat accumulation requires multicomponent and multilevel intervention [28]. Multicomponent intervention is effective in weight loss programs, which consist of changes that combine food choices, physical environments, sleep, and stress management, as described above (Figure 4). Furthermore, multilevel approaches focus on changing health behaviors by acting on multiple frameworks, including individual, interpersonal, organizational, community, and policy levels. Many intervention programs at the community level have been used to combat metabolic syndrome [28,38,39]. For instance, the healthy living program delivered by community coaching staff for overweight and obese me, who were football fans of the Scottish Premier League football clubs proved effective for weight loss of 4.94 kg (95% CI 3.95–5.94) after 12 months [39].

### 9.2. Healthcare Program by the Public System

Atherosclerotic cardiovascular diseases (ACVD) are life-threatening. Development of ACVD is often followed by serious complications. Therefore, it is important to detect asymptomatic cardiovascular risk factors and provide counter measures. For this purpose, a health check-up is a good opportunity to assess one’s own health status and assess the risk for cardiovascular diseases. In many countries, individuals are left on their own to decide whether to receive health check-ups or not. However, in the case of Japan, there is a universal public health screening program and a post-screening health educational system in place at the nation level to deal with metabolic syndrome, as described below [4,40]. There is also a disease management program (DMP) nationally in Germany. The Japanese intervention program aims at primary disease prevention, while the German DMP aims at secondary disease prevention (Figure 4) [41].

**Figure 5:**
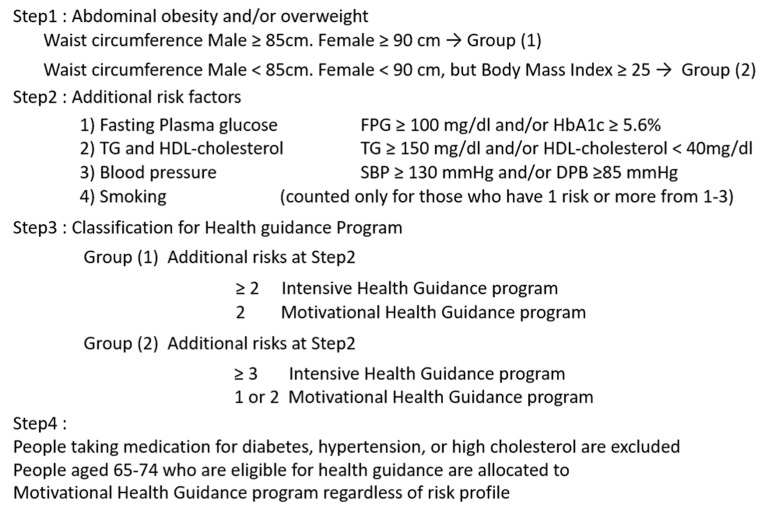
Participant classification for the Health Guidance program by the Ministry of Health, Labor, and Welfare in Japan [40].

The Japanese criteria for metabolic syndrome were established in 2005 [3,4], in which visceral fat accumulation was an essential component. In 2008, the Japanese government started a new screening and educational system for metabolic syndrome, focusing on visceral fat accumulation [40]. This new public health care system has the following features: (1) medical insurers are obliged to perform free health check-ups followed by health guidance to their subscribers aged 40 to 74 years; (2) methods of health check-ups and health guidance are standardized and health data are collected and assessed electronically; (3) subjects who need health guidance are stratified based on visceral fat accumulation and smoking habits (Figure 5). Participants who are thought to be “downstream” of metabolic syndrome are subjected to an intensive health guidance program with intermittent support over three months after the first interview followed by assessment six months later [40,42]. Recently, descriptive analysis in Japan has shown greater improvement in metabolic syndrome profiles in those individuals that participated in specific health guidance programs for three years than nonparticipants, although selection bias may be present [42]. Since it is difficult to set control conditions, there are only a few population-level or policy-level studies on health behaviors [42,43]. Further randomized control trials of health guidance programs are theoretically needed, which are now being undertaken in Japan [44].

### 9.3. Behavioral Approach Including Motivational Interviewing

In 2003, the U.S. Preventive Services Task Force recommended that primary care practitioners screen all adults for obesity and offer behavioral interventions and intensive counseling (Figure 4) [38]. Although a meta-analysis demonstrated that behavioral intervention resulted in a mean weight loss of 3.01 kg (95% CI: 4.02–2.01) [38], there are no established evidence-based guidelines for behavioral weight loss counseling in a primary care setting [28,38,45].

To encourage improvement of unhealthy lifestyles in health check-ups followed by health guidance, the following are important points: (1) individuals free of symptoms can assess their health status with regard to visceral fat accumulation to metabolic syndrome through what is called the “Where am I? chart” [46], for example using health data and pictorial presentation of carotid artery echograms; (2) individuals can go back to review their past and their own lifestyle using past health data, and understand the significance of reduction of accumulated visceral fat; (3) individuals can find out how to improve their lifestyle; and finally (4) individuals can appreciate improvement of health data, such as changes in blood glucose, lipid, and blood pressure, as well as weight loss and reduction of visceral fat in health check-ups to be conducted in subsequent years [42,46]. Behavioral modification in lifestyle by face-to-face individual counseling is important in reduction of visceral fat [40,42,46]. One recent innovation in improvement of face-to-face counseling is the implementation of online tools, including e-mail counseling and internet treatment programs (telemedicine) [47].

Health education (Figure 4) and preparation of food and physical environments (Figure 4) should be useful in facilitating behavioral modifications and practicing lifestyle improvements.

Since healthy living programs were reported to be effective for weight loss [39], recreational exercise might be useful as exercise therapy to combat metabolic syndrome. Recreational exercise and sports could be more feasible than the exercise therapy-based FITT (frequency, Intensity, time, type) principle, because recreation and sports are fun and encouraging for individuals. Therefore, recreational exercise and sports are suitable community-based strategies for physical activity and city planning [32].

It has been reported that on average, visceral fat accumulation is increasing in 20 to 30 year-old Japanese males, and exceeded 100 cm^2^ in 40-year old individuals [10]. Therefore, for prevention of metabolic syndrome, it is important to approach younger individuals. One of the targets of management of metabolic syndrome should be prevention of ACVD in the community. In the community, health education on lifestyle modification is important, and health guidance for acceleration of referral to physicians is also important for high-risk individuals [44]. In the medical field, medical intervention by focusing on visceral fat accumulation should be more efficient, since the etiology of metabolic diseases is diverse (Figure 2). Diabetic patients with visceral fat accumulation have dysregulated eating or sleeping behavior and progression of atherosclerosis [37,48]. Therefore, it is important to improve multiple metabolic diseases comprehensively by persistent lifestyle modification targeting reduction of visceral fat (Figure 1) [11,13,42]. On the other hand, an individual approach to each metabolic disease is needed for individuals with multiple risks without visceral fat accumulation (Figure 1; Figure 2) [49]. Programs against smoking, hypertension, and hypercholesterolemia are also quite important for individuals with or without visceral fat accumulation.

## 10. Conclusions

Taken together, the clinical significance of assessment of the metabolic syndrome is to link individuals with visceral fat–related multiple risk factors to follow health guidance, and to prevent atherosclerotic cardiovascular diseases by reducing visceral fat. To achieve this aim, in addition to personal approaches, population approaches are important to combat metabolic syndrome. Established evidence-based guidelines and programs are needed within primary care setting.

## Abbreviations

ACVD	atherosclerotic cardiovascular disease
BMI	body mass index
Mets	metabolic syndrome
SFA	subcutaneous fat area
VFA	visceral fat area
